# Occupational stress of physicians and nurses in emergency departments after contracting COVID-19 and its influencing factors: a cross-sectional study

**DOI:** 10.3389/fpubh.2023.1169764

**Published:** 2023-05-18

**Authors:** Chuanzhu Lv, Yong Gan, Jing Feng, Shijiao Yan, Heyu He, Xiaotong Han

**Affiliations:** ^1^Emergency Medicine Center, Sichuan Provincial People’s Hospital, University of Electronic Science and Technology of China, Chengdu, Sichuan, China; ^2^Research Unit of Island Emergency Medicine, Chinese Academy of Medical Sciences, Hainan Medical University, Haikou, Hainan, China; ^3^Key Laboratory of Emergency and Trauma of Ministry of Education, Hainan Medical University, Haikou, Hainan, China; ^4^Department of Social Medicine and Health Management, School of Public Health, Tongji Medical College, Huazhong University of Science and Technology, Wuhan, Hubei, China; ^5^Department of Emergency Medicine, Hunan Provincial People’s Hospital/The First Affiliated Hospital, Hunan Normal University, Changsha, Hunan, China; ^6^School of Public Health, Hainan Medical University, Haikou, Hainan, China; ^7^Department of Joint Surgery, The Second Affiliated Hospital of Hainan Medical University, Haikou, Hainan, China; ^8^The Emergency and Trauma College, Hainan Medical University, Haikou, Hainan, China; ^9^Clinical Research Center for Emergency and Critical Care in Hunan Province, Changsha, Hunan, China

**Keywords:** occupational stress, emergency department, physicians and nurses, COVID-19 infection, influencing factors

## Abstract

**Background:**

Occupational stress is one of the major occupational health hazards globally. This study investigated the current situation of and factors influencing the occupational stress of physicians and nurses in emergency departments (EDs) after contracting coronavirus disease (COVID-19).

**Methods:**

An online questionnaire survey was conducted among physicians and nurses in EDs in China between January 5 and 8, 2023. A general descriptive analysis of variables was conducted, the differences in the occupational stress of physicians and nurses in EDs with different characteristics were analyzed using the chi-square test, and factors influencing occupational stress were investigated using generalized ordinal logistic regression.

**Results:**

Of the 1924 physicians and nurses in EDs who contracted COVID-19, 64.71% considered their occupational stress high or very high, with overly intense work as the primary stressor. Those with ≥ 10 years of work tenure, working in tertiary hospitals and with higher professional titles were more stressed, while females, nurses, those with a master’s degree or higher, and those who continued to work after contracting COVID-19 were less stressed. There were differences in the predictors of occupational stress between physicians and nurses.

**Conclusion:**

China’s physicians and nurses in EDs had high occupational stress after contracting COVID-19. Attention should be given to the occupational mental health of physicians and nurses in EDs, and training on the prevention and treatment of COVID-19 infection should be strengthened.

## Introduction

Occupational stress is the physiological and psychological response that occurs when occupational demands exceed an individual’s capabilities (1). According to the World Health Organization, stress often occurs when employees perceive a lack of relevant knowledge and abilities to perform assigned tasks, low support from superiors and colleagues, or a loss of control over work processes (2). Occupational stress has been recognized as one of the major occupational health hazards that affects practitioners around the world (3), seriously endangering individuals’ physical and mental health (e.g., stroke and anxiety) (4, 5) and affecting organizational stability (e.g., absenteeism and turnover) (6, 7). Evidence suggests that various factors are linked to occupational stress, with common risk factors having been proven to be high work volume, long working hours, and high work intensity (8). In addition, occupational stress varies by sociodemographic characteristics, such as sex and education (9, 10).

Personnel in the medical field usually face a more stressful environment than those in other industries. The fact that health personnel in emergency departments (EDs) mainly treat critically ill patients and have a high workload leads to more pronounced occupational stress among physicians and nurses in these departments (8, 11). The daily need to make quick decisions in life-threatening situations, constant exposure to the death or suffering of patients, and the inability to provide adequate and appropriate medical care to patients greatly increase the burden on emergency health workers and contribute to a stressful work environment (12–14). Occupational stress among health workers in EDs should therefore be considered important.

The ongoing coronavirus disease (COVID-19) pandemic has placed a heavy burden on medical and healthcare systems around the world in the past three years. While the outbreak of the COVID-19 pandemic has changed the work environment for all employees, health workers must always be prepared to perform their duties. On December 7, 2022, the Chinese government promulgated the Notice on Further Optimizing the Implementation of Prevention and Control Measures for the COVID-19 Epidemic (15), proposing to further accurately divide risk areas, to avoid conducting nucleic acid testing of all populations by administrative region, and to scientifically classify and treat patients infected with COVID-19. EDs are important for responding to the COVID-19 epidemic and treating critically ill patients. After adjustment to the epidemic prevention policy, a surge of COVID-19 cases and an increase in the risk of infection have brought higher requirements and new challenges to physicians and nurses. Long working hours, an intense work environment, work overload, fear of COVID-19 infection, concerns about the health of family and friends, limited training and experience in the prevention and treatment of COVID-19 infection, and constant adjustments to and changes in treatment plans (16, 17) all have the potential to increase the stress of physicians and nurses in EDs.

A better understanding of the current status of occupational stress among physicians and nurses in EDs is conductive to developing targeted strategies for the improvement of mental health. However, to date, only very limited studies have been conducted to investigate the occupational stress of frontline physicians and nurses in EDs in the fight against the COVID-19 epidemic and the influencing factors (18–21), with the fear of contracting COVID-19 for themselves or their families being significantly associated with higher occupational health. Furthermore, research on the occupational stress of physicians and nurses in EDs after contracting COVID-19 and the influencing factors has not been reported. To fill this research gap, in this study, we aimed to investigate the current status of occupational stress of Chinese physicians and nurses in EDs who contracted COVID-19 and explore the influencing factors. This research is helpful to provide an empirical basis and suggestions for physicians and nurses in EDs to cope with occupational stress.

## Methods

### Study design and population

A cross-sectional study was conducted between January 5, 2023, and January 8, 2023. The convenience sampling method was used to recruit the study population. The Emergency Medicine Branch of the Chinese Medical Association organized an online conference on training and sharing experience in emergency medicine during the epidemic for physicians and nurses in EDs from 31 provinces/municipalities/autonomous regions in China (excluding Hong Kong, Macau, and Taiwan) from December 25, 2022, to January 6, 2023. This special training conference was conducted in a voluntary manner. We distributed an electronic questionnaire to participants through the conference WeChat group on January 5, 2023, collected responses on a voluntary basis, and closed the questionnaire submission system on January 8, 2023. A total of 2,447 questionnaires were collected, with respondents covering 29 provinces/municipalities/autonomous regions except Qinghai and Tibet. The questionnaires for six nonemergency physicians and nurses were excluded. The remaining 1,924 confirmed samples were further screened based on whether they were infected with COVID-19 between December 1, 2022, and the time of the questionnaire survey.

This study was approved by the Ethics Committee of Hainan Medical College (No. HYLL-2022-426), and all participants provided informed consent and voluntarily participated in the investigation.

### Measurement

#### Dependent variable

Although some occupational stress scales have been reported and confirmed the validity in the previously published literature (22–24), no specific standardized questionnaire or scale is available for health workers in EDs in China. Additionally, because health workers in EDs experienced a heavy workload during the COVID-19 pandemic in China, the time required to complete the questionnaire should be shortened as much as possible. Thus, with reference to a previous study among emergency medical personnel during the COVID-19 pandemic (18), occupational stress was measured using the following item: “Have you felt stressed at work recently?” Responses of “no stress,” “low stress,” “average stress,” “high stress,” and “very high stress” were scored 1, 2, 3, 4, and 5 points, respectively. Based on literature reviews (8, 17–20) and our team’s own research experiences during the COVID-19 pandemic, a multiple-choice item with eight options was used to further investigate stressors. The options were as follows: (1) worried about being infected and unable to go to work; (2) concerned about having been infected and feeling very fatigued at work; (3) work intensity is too high; (4) too many severely ill patients; (5) work environment is depressing; (6) current knowledge and skills fail to meet the needs of patients; (7) worried about COVID-19-infected family members and failed to balance work and family; and (8) other.

#### Independent variables

The independent variables in this study were mainly sociodemographic characteristics and work characteristics, including age, years of work tenure, sex, occupation, highest education level, hospital level, professional title, and work status after contracting COVID-19.

#### Quality control

The questionnaire was designed based on literature research (8, 17–20), expert consultations, and a group discussion. First, a literature review was performed and we developed a questionnaire. Next, two physicians practicing in EDs, two nurses practicing in EDs, two healthcare administrators practicing in hospitals, a social medicine professor, and an epidemiology professor with at least five years of work experience were selected to assess the questionnaire content. The main suggestion given by the experts was to refine the questionnaire to reduce the completion time. Then, six members of our research team conducted a group discussion to clarify the instructions of the questionnaire and its distribution. To ensure that all questions were clear and understandable, a pre-survey of 30 physicians and nurses in EDs in Haikou was conducted before the formal survey. Some statements in the instructions of the questionnaire were found to be vague, and the questionnaire was further revised based on their feedback. The survey was then conducted electronically using Wenjuanxing, a Chinese online questionnaire survey platform, with the help of the Emergency Medicine Branch of the Chinese Medical Association. The same device or account could only be used to complete the questionnaire once, and all questions had to be answered before submission.

#### Statistical analysis

Data analysis was performed using Stata 17.0. Quantitative variables were described as means and standard deviations, and categorical variables were presented as frequencies and constituent ratios. The chi-square test was used to compare the differences in occupational stress for different variables. The statistically significant variables were included in an ordinal logistic regression model of the factors influencing occupational stress and did not pass the parallelism test. Therefore, a generalized ordinal logistic regression analysis that satisfied the partial proportional odds assumption was performed using the gologit2 command (25). The dependent variable, occupational stress, was a categorical ordinal variable with five levels and led to four logit models, namely (1), 2, 3, 4, and 5 vs. 1 (2), 3, 4, and 5 vs. 1 and 2 (3), 4 and 5 vs. 1, 2, and 3, and (4) 5 vs. 1, 2, 3, and 4, which were generated to compare the probability of being in a higher category with the probability of being below that category. Furthermore, a stratified analysis was conducted to identify the determinants of occupational stress among physicians and nurses in EDs. Differences were considered statistically significant at a two-sided threshold of *p* < 0.05.

## Results

The 1,924 physicians and nurses in EDs had a mean age of 40.49 ± 4.93 years, with the majority (84.62%) under 45 years. The respondents had worked for a mean of 12.91 ± 6.44 years, with the majority (64.55%) having a work tenure of 10 years or more. Males and females accounted for 48.34 and 51.66%, respectively, physicians and nurses accounted for 57.69 and 42.31%, respectively, more than half (51.61%) had a bachelor’s degree, approximately two-thirds (66.63%) worked in tertiary hospitals, many (46.93%) had junior professional titles and below, and nearly one-fifth continued to work after contracting COVID-19. Details are shown in Table 1.

**Table 1:** Descriptive statistics and univariate analysis of the differences in occupational stress among physicians and nurses in the emergency department.

Variables	Frequency (%)	Occupational stress					*χ^2^*
		1	2	3	4	5	
Total	1924 (100.00)	161 (8.37)	228 (11.85)	290 (15.07)	622 (32.33)	623 (32.38)	
Age, years							30.38^*^
< 45	1,628 (84.62)	147 (9.03)	211 (12.96)	254 (15.60)	519 (31.88)	497 (30.53)	
≥ 45	296 (15.38)	14 (4.73)	17 (5.74)	36 (12.16)	103 (34.80)	126 (42.57)	
Work tenure, years							300.63^*^
< 10	682 (35.45)	103 (15.10)	151 (22.14)	158 (23.17)	134 (19.65)	136 (19.94)	
≥ 10	1,242 (64.55)	58 (4.67)	77 (6.20)	132 (10.63)	488 (39.29)	487 (39.21)	
Sex							28.59^*^
Male	930 (48.34)	60 (6.45)	84 (9.03)	141 (15.16)	308 (33.12)	337 (36.24)	
Female	994 (51.66)	101 (10.16)	144 (14.49)	149 (14.99)	314 (31.59)	286 (28.77)	
Occupation							31.34^*^
Physician	1,110 (57.69)	68 (6.13)	121 (10.90)	167 (15.05)	350 (31.53)	404 (36.40)	
Nurse	814 (42.31)	93 (11.43)	107 (13.14)	123 (15.11)	272 (33.42)	219 (26.90)	
Education level							116.13^*^
Associate’s degree or vocational diploma^#^	491 (25.52)	54 (11.00)	48 (9.78)	75 (15.27)	163 (33.20)	151 (30.75)	
Bachelor’s degree	993 (51.61)	62 (6.24)	95 (9.57)	106 (10.67)	366 (36.86)	364 (36.66)	
Master’s degree or higher	440 (22.87)	45 (10.23)	85 (19.32)	109 (24.77)	93 (21.14)	108 (24.55)	
Level of hospital							88.00^*^
Others	105 (5.46)	14 (13.33)	24 (22.86)	28 (26.67)	21 (20.00)	18 (17.14)	
Secondary hospital	537 (27.91)	61 (11.36)	88 (16.39)	95 (17.69)	136 (25.33)	157 (29.24)	
Tertiary hospital	1,282 (66.63)	86 (6.71)	116 (9.05)	167 (13.03)	465 (36.27)	448 (34.95)	
Professional title							29.14^*^
Elementary or below	903 (46.93)	80 (8.86)	114 (12.62)	141 (15.61)	318 (35.22)	250 (27.69)	
Intermediate	620 (32.22)	46 (7.42)	61 (9.84)	90 (14.52)	208 (33.55)	215 (34.68)	
Senior	401 (20.84)	35 (8.73)	53 (13.22)	59 (14.71)	96 (23.94)	158 (39.40)	
Continued working after contracting COVID-19							271.18^*^
No	1,540 (80.04)	91 (5.91)	128 (8.31)	189 (12.27)	557 (36.17)	575 (37.34)	
Yes	384 (19.96)	70 (18.23)	100 (26.04)	101 (26.30)	65 (16.93)	48 (12.50)	

* *p* < 0.05.

# Physicians and nurses in the emergency department who have acquired associate’s degrees or vocational diplomas. An associate degree requires 3 years of education in college after graduation from senior middle school (grade year 10 to year 12), or 5 years of education in college after graduation from junior middle school (grade year 7 to year 9). A vocational diploma requires 2 years of education in vocational schools after graduation from senior middle school, or 3 years of education in vocational schools after graduation from junior middle school.

Only 8.37% indicated that they had “no stress,” and those with “low stress,” “average stress,” “high stress,” and “very high stress” accounted for 11.85, 15.07, 32.33, and 32.38%, respectively. The chi-square test showed a statistically significant distribution (*p* < 0.05) of occupational stress among physicians and nurses in EDs in terms of age, years of work tenure, sex, occupation, highest education level, hospital rank, professional title, and whether they continued to work after contracting COVID-19 (Table 1). Table 2 presents the sources of occupational stress of physicians and nurses in EDs, with overly intense work (77.08%), contracting COVID-19 themselves, being very fatigued at work (69.82%), and too many critically ill patients (62.00%) being the 3 main stressors.

**Table 2:** Distribution of the source of occupational stress among physicians and nurses in the emergency department.

Items	*N*	%
Total	1763	100.00
Worried about getting infected and not going to work	677	38.40
Having been infected with COVID-19 and especially tired at work	1,231	69.82
Intensive work	1,359	77.08
Too many critical patients	1,093	62.00
Depressing work environment	916	51.96
Knowledge and skills cannot meet the needs of patients	542	30.74
Unable to juggle work and family due to family members being infected with COVID-19	873	49.52
Other	17	0.96

Table 3 shows the results of the generalized ordinal logistic regression of the factors influencing the occupational stress of physicians and nurses in EDs. In all models, years of work tenure, occupation, and whether they continued to work after contracting COVID-19 were all statistically significant. Work tenure of 10 years or longer was a risk factor for occupational stress, and its effect increased when the occupational stress level moved from low to high. The largest effect was identified in Model 3 (*b* = 1.09, *p* < 0.01), and decreased in Model 4. Working as a nurse and continuing to work after contracting COVID-19 were protective factors against occupational stress. The effect of occupation weakened when the occupational stress level moved from low to high; the smallest effect was identified in Model 3 (b = −0.30, *p* = 0.03), and increased in Model 4. The effect of whether to continue working after contracting COVID-19 strengthened when the occupational stress level moved from low to high; the largest effect was identified in Model 3 (*b* = −1.28, *p* < 0.01), and decreased in Model 4. The highest education level and hospital level were not statistically significant in Model 1 but were statistically significant in Models 2 to 4. Individuals with a master’s degree or higher had less occupational stress, and those working in tertiary hospitals had more occupational stress. Furthermore, in Models 2 and 3, occupational stress was significantly lower for females than for males. Model 4 shows that the higher the professional title, the greater the occupational stress. However, age was not significantly associated with occupational stress of physicians and nurses in EDs.

**Table 3:** Generalized ordered logistic regression model for the factors associated with occupational stress among physicians and nurses in the emergency department.

Variables	Model 1: 2, 3, 4, 5 vs. 1			Model 2: 3, 4, 5 vs. 1, 2			Model 3: 4, 5 vs. 1, 2, 3			Model 4: 5 vs. 1, 2, 3, 4		
	*b*	95% CI	*z*	*b*	95% CI	*z*	*b*	95% CI	*z*	*b*	95% CI	*z*
Age, years (ref: < 45)												
≥ 45	0.31	−0.29, 0.91	1.03	0.30	−0.13, 0.73	1.35	0.20	−0.13, 0.53	1.17	0.00	−0.29, 0.30	0.03
Work tenure, years (ref: < 10)												
≥ 10	0.73	0.37, 1.09	3.98^*^	1.00	0.73, 1.26	7.37^*^	1.09	0.85, 1.33	9.00^*^	0.42	0.16, 0.68	3.14^*^
Sex (ref: male)												
Female	−0.14	−0.53, 0.25	−0.71	−0.38	−0.66, −0.10	−2.65^*^	−0.25	−0.50, −0.01	−2.03^*^	−0.05	−0.31, 0.20	−0.42
Occupation (ref: physician)												
Nurse	−0.50	−0.98, −0.01	−2.02^*^	−0.43	−0.74, −0.11	−2.67^*^	−0.30	−0.57, −0.03	−2.14^*^	−0.35	−0.63, −0.07	−2.41^*^
Education level (ref: associate’s degree or vocational diploma^#^)												
Bachelor’s degree	0.38	−0.04, 0.81	1.77	0.08	−0.23, 0.39	0.50	0.30	0.04, 0.56	2.28^*^	0.08	−0.17, 0.32	0.62
Master’s degree or higher	−0.02	−0.61, 0.58	−0.05	−0.54	−0.92, −0.15	−2.73^*^	−0.62	−0.95, −0.29	−3.69^*^	−0.40	−0.74, −0.06	−2.29^*^
Level of hospital (ref: others)												
Secondary hospital	0.03	−0.61, 0.67	0.09	0.20	−0.27, 0.67	0.83	0.47	0.00, 0.93	1.98^*^	0.42	−0.14, 0.98	1.47
Tertiary hospital	0.38	−0.24, 1.01	1.21	0.68	0.23, 1.13	2.96^*^	0.99	0.55, 1.44	4.38^*^	0.66	0.12, 1.20	2.40^*^
Professional title (ref: elementary or below)												
Intermediate	0.06	−0.33, 0.45	0.28	0.09	−0.19, 0.38	0.66	0.02	−0.22, 0.26	0.15	0.26	0.03, 0.49	2.21^*^
Senior	−0.07	−0.50, 0.36	−0.31	−0.15	−0.46, 0.17	−0.92	−0.11	−0.39, 0.17	−0.74	0.55	0.26, 0.85	3.70^*^
Whether to continue working after contracting COVID-19 (ref: no)												
Yes	−0.80	−1.14, −0.45	−4.52^*^	−1.05	−1.31, −0.78	−7.85^*^	−1.28	−1.54, −1.02	−9.50^*^	−1.20	−1.54, −0.85	−6.80^*^
Constant	2.09	1.25, 2.92	4.91^*^	1.11	0.53, 1.68	3.77^*^	−0.29	−0.82, 0.25	−1.05	−1.42	−2.04, −0.81	−4.56^*^

* *p* < 0.05.

# Physicians and nurses in the emergency department who have acquired associate’s degrees or vocational diplomas. An associate degree requires 3 years of education in college after graduation from senior middle school (grade year 10 to year 12), or 5 years of education in college after graduation from junior middle school (grade year 7 to year 9). A vocational diploma requires 2 years of education in vocational schools after graduation from senior middle school, or 3 years of education in vocational schools after graduation from junior middle school.

The results of stratified generalized ordinal logistic regression show that the predictors of occupational stress varied between physicians and nurses in EDs (Appendix Table S1). Work tenure, education level, level of hospital, professional title, and whether individuals continued working after contracting COVID-19 were common factors associated with occupational stress of physicians and nurses. Nevertheless, the effect of education level and professional title on occupational stress was opposite between physicians and nurses in EDs; specifically, higher education levels indicated lower occupational stress in physicians and higher occupational stress in nurses, while higher professional titles indicated higher occupational stress in physicians and lower occupational stress in nurses. Age and sex were another two factors associated with occupational stress among physicians in EDs.

## Discussion

This study found that 91.63% of physicians and nurses in EDs felt stressed at work after contracting COVID-19 and that nearly two-thirds felt highly and very highly stressed. In the context of the COVID-19 pandemic, previous studies have reported a lower incidence of stress. Çınar et al. (20) surveyed 169 emergency department nurses in Turkey and found that 44.6% had higher than average perceived stress. Cui et al. (19) analyzed 453 EDs and fever clinics in Jiangsu Province, China, and reported that 32.23% of nurses had high stress. A meta-analysis revealed that frontline medical workers who cared for COVID-19 patients had a stress incidence of 45% (26). Differences in stress levels may be related to COVID-19 infection status, study area, samples, and measurement tools. Overall, physicians and nurses who contracted COVID-19 in Chinese EDs were under high occupational stress. The COVID-19 pandemic affected the stress levels of physicians and nurses in EDs, made them work under stressful conditions, and increased the risk of psychological problems (17). Hospital administrators should pay close attention to the stress of physicians and nurses in EDs, train them to cope with the COVID-19 epidemic, and improve their mental health.

The primary source of occupational stress among physicians and nurses in EDs was overly high work intensity. An excessive number of critically ill patients was also an important stressor, indicating that physicians and nurses in EDs had a high workload. After adjustments to the epidemic prevention policy, the number of COVID-19 cases increased sharply as did the demand for medical treatment, thereby increasing the workload of personnel at EDs. Furthermore, the infection of medical workers led to a shortage of human resources in EDs, making it difficult to keep up with the supply of medical services; therefore, the medical personnel on duty were overworked. A survey by Şanlıtürk et al. (27) of intensive care nurses during the COVID-19 pandemic found similar results, with 78.6% of nurses reporting that stress stemmed from a heavy workload and prolonged fatigue. Mirzaei et al. (21) found that the highest level of job stress was related to the demand area among ED nurses and emergency medical services staff, and increasing the workload led to job stress. The COVID-19 pandemic changed the functioning of hospitals and specialist clinics, especially burdening the already overloaded health workforce in EDs. It is necessary to strengthen the overall planning of medical resources, coordinate work shifts, and mobilize physicians and nurses from other departments to participate in emergency treatment when necessary.

Having contracted COVID-19 themselves and being very fatigued at work were important sources of occupational stress, an option chosen by 69.82% of physicians and nurses in EDs. In addition, multivariate analysis showed that continuing to work after contracting COVID-19 was a protective factor against occupational stress. In this study, 19.96% of physicians and nurses in EDs continued to work after contracting COVID-19, indicating that the physicians and nurses in EDs who had contracted COVID-19 but continued work despite feeling fatigued during work were more resilient to stress. Because this was a cross-sectional study, a causal relationship could not be determined, and it is possible that individuals with less occupational stress tended to continue to work despite being infected.

Previous studies on years of work tenure or sex differences in the occupational stress of medical workers have led to inconsistent results. Povedano-Jimenez et al. (28) noted that males with more than 10 years of work tenure showed greater coping skills in difficult and stressful situations. In contrast, a study by Tian et al. (29) on Chinese emergency physicians showed that male sex and long work tenure were positively correlated with high occupational stress. However, Mirzaei et al. (21) reported that gender and work experience were not significant factors that affected the occupational stress of ED nurses and emergency medical services staff. Our study found that work tenure of 10 years or longer was a risk factor for occupational stress. COVID-19 is an emerging infectious disease, and previous skills and clinical experience in emergency medicine acquired over time may not be applicable in the response to the COVID-19 epidemic. Hence, work experience played a limited role. In addition, the results of this study revealed that females had less occupational stress than males. In contrast, most previous studies have shown that occupational stress was more prevalent among female medical workers, who were more affected by the double burden from both family and work (18, 20, 27). One possible explanation for our finding may be that women received dual psychological support despite being affected by work and family disturbances. Further research is needed on the relationship between sex and occupational stress.

The generalized ordinal logistic regression analysis results showed that in EDs, physicians had significantly higher occupational stress than nurses. There are differences between physicians and nurses in the nature of their work; physicians are mainly responsible for diagnosing diseases and developing treatment plans, and nurses mainly play a supportive role by carrying out physicians’ plans. As a result, patients and their families have higher expectations of physicians, which may increase the stress of physicians to some extent. Studies have shown that physicians were more likely to report adverse psychological consequences of occupational stress than nurses (30, 31). Therefore, focus should be placed on physicians to provide them with adequate psychological support, develop their resilience to stress, and offer timely interventions when psychological problems are identified.

The results of stratified analysis indicated that the predictors of occupational stress differed between physicians and nurses in EDs. Intriguingly, education level and professional title had opposite effects on occupational stress between physicians and nurses. Physicians with lower education levels and higher professional titles reported higher occupational stress. A low level of education usually indicates a lack of competence to cope with the diagnosis and treatment of diseases, and patients prefer to seek higher-quality health care from those who have higher professional titles (32), both of which may contribute to a higher level of occupational stress among physicians. For nurses, higher education levels and lower professional titles were associated with higher occupational stress. Although higher education increases an individual’s knowledge and skills, it is expected to improve the quality of health services with the growth of people’s expectations (33). Better-educated people are often in more challenging situations and perform more specialized work, increasing the level of occupational stress (34). The role of nurses with low professional titles was limited during the COVID-19 pandemic, which may have caused them to lack a sense of presence and led to stress. More research is needed to explore the determinants of occupational stress among physicians and nurses in EDs.

## Strengths and limitations

This study is the first to investigate the occupational stress of physicians and nurses in EDs after they contracted COVID-19 and to analyze the stressors and influencing factors. The findings may serve as a reference for other countries and other groups of medical workers. Notably, this study had some limitations. First, the cross-sectional study design limited causal inferences. Second, the collection of self-reported data may reduce the objectivity of the information. Third, there may be other influencing factors (e.g., psychological factors and workload) that were not examined. Fourth, convenience sampling limited the representativeness of the sample and the generalizability of our findings.

### Implications for research and practice

To better deliver medical and healthcare services during the COVID-19 pandemic, it is vital to protect the physical and mental health of physicians and nurses. The results of this study suggest that we should pay close attention to the psychological status of physicians and nurses, strengthen training for COVID-19 diagnosis and treatment, and flexibly allocate medical resources. This study provides scientific evidence for the research and management of the occupational stress of physicians and nurses in EDs and offers a reference for the management of occupational stress of physicians and nurses under similar public health emergencies.

## Conclusion

Chinese physicians and nurses in EDs had a high level of occupational stress after contracting COVID-19, with heavy workloads and fatigue at work after infection as the main stressors. Age, years of work tenure, sex, occupation, education level, hospital level, professional title, and continuing to work after contracting COVID-19 were the factors that influenced the occupational stress of physicians and nurses in EDs.

## Data availability statement

The raw data supporting the conclusions of this article will be made available by the authors, without undue reservation.

## Ethics statement

The studies involving human participants were reviewed and approved by the Ethics Committee of Hainan Medical College (No. HYLL-2022-426). All participants provided informed consent and voluntarily participated in the investigation. The patients/participants provided their verbal informed consent to participate in this study.

## Author contributions

CL and YG conceived and designed the study. CL, SY, HH, and XH participated in the acquisition of data. CL and JF analyzed the data. HH and XH gave advice on methodology. YG and JF wrote the draft of the paper. XH is the guarantor of this work and has full access to all the data in the study and takes responsibility for its integrity and the accuracy of the data analysis. All authors contributed to writing, reviewing, or revising the paper and read and approved the final manuscript.

## Funding

This study was supported by the National Natural Science Foundation of China (8216120150), Key Laboratory of Emergency and Trauma of Ministry of Education (Hainan Medical University) (Grant. KLET-202101), and Key Laboratory of Emergency and Trauma of Ministry of Education (Hainan Medical University) (Grant. KLET-202103).

## Conflict of interest

The authors declare that the research was conducted in the absence of any commercial or financial relationships that could be construed as a potential conflict of interest.

## Publisher’s note

All claims expressed in this article are solely those of the authors and do not necessarily represent those of their affiliated organizations, or those of the publisher, the editors and the reviewers. Any product that may be evaluated in this article, or claim that may be made by its manufacturer, is not guaranteed or endorsed by the publisher.

## Abbreviations

COVID-19: coronavirus disease
EDs: emergency departments
