# Supplementary material for: Occupational stress of physicians and nurses in emergency departments after contracting COVID-19 and its influencing factors: a cross-sectional study
Source: Front Public Health. 2023 May 18;11:1169764. doi: 10.3389/fpubh.2023.1169764 (PMC10232976; doi:10.3389/fpubh.2023.1169764)
Supplement: Supplementary file 1 [file Table_1.doc]

**TABLE 1 The stratified analysis of the predictors of occupational stress among physicians and nurses in the emergency department**

| Variables | Model 1: 2, 3, 4, 5 vs. 1 | | | Model 2: 3, 4, 5 vs. 1, 2 | | | Model 3: 4, 5 vs. 1, 2, 3 | | | Model 4: 5 vs. 1, 2, 3, 4 | | |
| --- | --- | --- | --- | --- | --- | --- | --- | --- | --- | --- | --- | --- |
|  | b | 95% CI | z | b | 95% CI | z | b | 95% CI | z | b | 95% CI | z |
| Physician |  |  |  |  |  |  |  |  |  |  |  |  |
| Age, years (ref: < 45) |  |  |  |  |  |  |  |  |  |  |  |  |
| ≥ 45 | 1.06 | -0.08, 2.20 | 1.82 | 0.81 | 0.14, 1.48 | 2.38^*^ | 0.40 | -0.07, 0.86 | 1.67 | 0.05 | -0.33, 0.42 | 0.24 |
| Work tenure, years (ref: < 10) |  |  |  |  |  |  |  |  |  |  |  |  |
| ≥ 10 | 0.58 | 0.02, 1.14 | 2.03^*^ | 0.57 | 0.18, 0.96 | 2.88^*^ | 0.78 | 0.43, 1.12 | 4.44^*^ | 0.23 | -0.14, 0.60 | 1.24 |
| Sex (ref: male) |  |  |  |  |  |  |  |  |  |  |  |  |
| Female | 0.25 | -0.32, 0.83 | 0.86 | -0.56 | -0.92, -0.21 | -3.11^*^ | -0.46 | -0.77, -0.16 | -2.96^*^ | -0.20 | -0.52, 0.11 | -1.26 |
| Education level (ref: associate’s degree or vocational diploma^#^) | | | |  |  |  |  |  |  |  |  |  |
| Bachelor’s degree | -0.22 | -1.88, 1.44 | -0.26 | -1.41 | -2.47, -0.35 | -2.61^*^ | -0.55 | -1.11, 0.00 | -1.95 | -0.21 | -0.58, 0.17 | -1.09 |
| Master’s degree or higher | -0.86 | -2.51, 0.78 | -1.03 | -2.30 | -3.35, -1.24 | -4.25^*^ | -1.49 | -2.08, -0.91 | -5.01^*^ | -0.84 | -1.31, -0.38 | -3.57^*^ |
| Level of hospital (ref: others) |  |  |  |  |  |  |  |  |  |  |  |  |
| Secondary hospital | -0.48 | -1.73, 0.77 | -0.75 | 0.21 | -0.49, 0.90 | 0.58 | 0.65 | -0.01, 1.30 | 1.94 | 0.34 | -0.38, 1.07 | 0.92 |
| Tertiary hospital | -0.32 | -1.55, 0.90 | -0.52 | 0.62 | -0.05, 1.30 | 1.80 | 0.95 | 0.32, 1.59 | 2.93^*^ | 0.51 | -0.20, 1.22 | 1.42 |
| Professional title (ref: elementary or below) | | |  |  |  |  |  |  |  |  |  |  |
| Intermediate | 0.53 | -0.07, 1.14 | 1.72 | 0.25 | -0.15, 0.66 | 1.23 | 0.26 | -0.07, 0.59 | 1.54 | 0.41 | 0.11, 0.72 | 2.63^*^ |
| Senior | 0.26 | -0.37, 0.90 | 0.81 | 0.26 | -0.18, 0.70 | 1.17 | 0.28 | -0.09, 0.65 | 1.51 | 0.91 | 0.53, 1.29 | 4.72^*^ |
| Whether to continue working after contracting COVID-19 (ref: no) | | | | |  |  |  |  |  |  |  |  |
| Yes | -0.75 | -1.31, -0.20 | -2.68^*^ | -0.93 | -1.31, -0.55 | -4.80^*^ | -1.23 | -1.61, -0.86 | -6.48^*^ | -1.03 | -1.49, -0.56 | -4.34^*^ |
| Constant | 3.04 | 0.95, 5.12 | 2.85^*^ | 2.71 | 1.47, 3.95 | 4.28^*^ | 0.50 | -0.34, 1.33 | 1.17 | -1.01 | -1.83, -0.18 | -2.40^*^ |
| Variables | Model 5: 2, 3, 4, 5 vs. 1 | | | Model 6: 3, 4, 5 vs. 1, 2 | | | Model 7: 4, 5 vs. 1, 2, 3 | | | Model 8: 5 vs. 1, 2, 3, 4 | | |
|  | b | 95% CI | z | b | 95% CI | z | b | 95% CI | z | b | 95% CI | z |
| Nurses |  |  |  |  |  |  |  |  |  |  |  |  |
| Age, years (ref: < 45) |  |  |  |  |  |  |  |  |  |  |  |  |
| ≥ 45 | -0.26 | -1.01, 0.49 | -0.67 | -0.25 | -0.83, 0.34 | -0.83 | -0.04 | -0.55, 0.46 | -0.16 | -0.13 | -0.65, 0.39 | -0.48 |
| Work tenure, years (ref: < 10) |  |  |  |  |  |  |  |  |  |  |  |  |
| ≥ 10 | 0.67 | 0.20, 1.14 | 2.77^*^ | 1.11 | 0.74, 1.49 | 5.84^*^ | 1.18 | 0.84, 1.53 | 6.72^*^ | 0.40 | -0.01, 0.81 | 1.90 |
| Sex (ref: male) |  |  |  |  |  |  |  |  |  |  |  |  |
| Female | -0.33 | -1.00, 0.35 | -0.95 | -0.05 | -0.54, 0.44 | -0.20 | 0.04 | -0.41, 0.50 | 0.19 | 0.19 | -0.35, 0.74 | 0.70 |
| Education level (ref: associate’s degree or vocational diploma^#^) | | | |  |  |  |  |  |  |  |  |  |
| Bachelor’s degree | 0.43 | -0.02, 0.88 | 1.89 | 0.21 | -0.14, 0.55 | 1.15 | 0.51 | 0.19, 0.83 | 3.11^*^ | 0.16 | -0.19, 0.50 | 0.89 |
| Master’s degree or higher | 14.28 | -870.81, 899.36 | 0.03 | 1.53 | 0.45, 2.62 | 2.76^*^ | 0.09 | -0.57, 0.75 | 0.27 | 0.44 | -0.25, 1.13 | 1.25 |
| Level of hospital (ref: others) |  |  |  |  |  |  |  |  |  |  |  |  |
| Secondary hospital | 0.26 | -0.53, 1.05 | 0.64 | 0.12 | -0.53, 0.78 | 0.36 | 0.27 | -0.41, 0.94 | 0.77 | 0.58 | -0.36, 1.51 | 1.21 |
| Tertiary hospital | 0.75 | -0.02, 1.52 | 1.92 | 0.60 | -0.03, 1.22 | 1.88 | 1.02 | 0.39, 1.66 | 3.15^*^ | 0.95 | 0.06, 1.84 | 2.09^*^ |
| Professional title (ref: elementary or below) | | |  |  |  |  |  |  |  |  |  |  |
| Intermediate | -0.32 | -0.83, 0.20 | -1.19 | 0.02 | -0.38, 0.42 | 0.11 | -0.20 | -0.55, 0.15 | -1.15 | 0.14 | -0.21, 0.49 | 0.79 |
| Senior | -0.37 | -0.98, 0.23 | -1.22 | -0.51 | -0.99, -0.04 | -2.12^*^ | -0.55 | -1.02, -0.09 | -2.32^*^ | 0.00 | -0.55, 0.56 | 0.01 |
| Whether to continue working after contracting COVID-19 (ref: no) | | | | |  |  |  |  |  |  |  |  |
| Yes | -0.64 | -1.10, -0.18 | -2.71^*^ | -0.93 | -1.30, -0.55 | -4.87^*^ | -1.22 | -1.60, -0.83 | -6.20^*^ | -1.32 | -1.87, -0.77 | -4.73^*^ |
| Constant | 1.50 | 0.49, 2.51 | 2.90^*^ | 0.34 | -0.45, 1.13 | 0.84 | -0.88 | -1.67, -0.09 | -2.18^*^ | -2.21 | -3.26, -1.15 | -4.10^*^ |

^*^*P* < 0.05.

^#^Physicians and nurses in the emergency department who have acquired associate’s degrees or vocational diplomas. An associate degree requires 3 years of education in college after graduation from senior middle school (grade year 10 to year 12), or 5 years of education in college after graduation from junior middle school (grade year 7 to year 9). A vocational diploma requires 2 years of education in vocational schools after graduation from senior middle school, or 3 years of education in vocational schools after graduation from junior middle school.
